# A new clinical tool for assessing numerical abilities in neurological diseases: numerical activities of daily living

**DOI:** 10.3389/fnagi.2014.00112

**Published:** 2014-06-20

**Authors:** Carlo Semenza, Francesca Meneghello, Giorgio Arcara, Francesca Burgio, Francesca Gnoato, Silvia Facchini, Silvia Benavides-Varela, Maurizio Clementi, Brian Butterworth

**Affiliations:** ^1^Dipartimento di Neuroscienze, Università di PadovaPadova, Italy; ^2^Laboratorio di Neuropsicologia, I.R.C.C.S. Fondazione Ospedale San CamilloLido di Venezia, Italy; ^3^Dipartimento di Psicologia Generale, Università di PadovaPadova, Italy; ^4^Dipartimento Salute Donna e Bambino, Università di PadovaPadova, Italy; ^5^Institute of Cognitive Neuroscience, University College LondonUK

**Keywords:** acalculia, activities of daily living, mathematical cognition, neurological diseases, neuropsychological assessment

## Abstract

The aim of this study was to build an instrument, the numerical activities of daily living (NADL), designed to identify the specific impairments in numerical functions that may cause problems in everyday life. These impairments go beyond what can be inferred from the available scales evaluating activities of daily living in general, and are not adequately captured by measures of the general deterioration of cognitive functions as assessed by standard clinical instruments like the MMSE and MoCA. We assessed a control group (*n* = 148) and a patient group affected by a wide variety of neurological conditions (*n* = 175), with NADL along with IADL, MMSE, and MoCA. The NADL battery was found to have satisfactory construct validity and reliability, across a wide age range. This enabled us to calculate appropriate criteria for impairment that took into account age and education. It was found that neurological patients tended to overestimate their abilities as compared to the judgment made by their caregivers, assessed with objective tests of numerical abilities.

## Introduction

Number processing and calculation are an essential part of our culture. We use numbers for counting, measuring, comparing, putting things in order, etc. Moreover, we constantly need to calculate, understand fractions, proportions and ratios, and to understand and remember PIN codes, telephone numbers, addresses, shoe sizes, and so on. Of course, many occupations need relatively high levels of numerical skill, and poor skills will have an adverse effect on life chances in education and employment, indeed a larger effect than poor literacy skills, as a large-scale cohort study in the UK demonstrates (Parsons and Bynner, 2005).

In the past two decades, neuroscience has made a significant progress in the understanding of how the brain represents numerical information and sustains mathematical computation (Butterworth and Walsh, 2011). One of the main sources of evidence has been the observation of patients, with acute, stable or progressive brain damage that gives rise to a range of specific disorders of number processing and calculation, usually referred to as “acquired acalculia” (Hecaen and Angelergues, 1961; Semenza, 2008; Willmes, 2008; Ward, 2010).

Despite the many studies of the neural basis of typical and atypical mathematical abilities the impact of such important disturbances on an individual's everyday life is still unclear. Deterioration of mathematical abilities is a very frequent consequence of brain damage, however, and a socially relevant one. In the elderly, even in healthy ones, slowing of arithmetical functions is found, which is often clinically hard to distinguish from that produced by neurological deterioration (Zamarian et al., 2007). Moreover, slowing of numerical skills with age may have many causes, not just degenerative diseases and evaluating this deterioration will be preliminary to effective retraining.

Research with brain-damaged patients has repeatedly demonstrated a range of quite specific deficits. For example, there may be selective deficits in number transcoding from spoken to written numbers, or from written to spoken numbers, arithmetical signs, arithmetical facts and rules, arithmetical procedures and conceptual knowledge have been shown to be selectively disrupted after brain damage (see for reviews Butterworth, 1999; Cipolotti and van Harskamp, 2001; Semenza et al., 2006; Semenza, 2008). For instance, some patients were unable to use numbers in the Arabic code but could use the alphabetical code (Cipolotti et al., 1994). Patients may also be found with impairments in one type of operation and not in others, such as addition but not multiplication, or subtraction but not addition (Cipolotti and van Harskamp, 2001). Some of these cases are prima facie counterintuitive, like the case of sparing of division relative to multiplication (Venneri and Semenza, 2011). Impaired math skills can coexist with apparently normal reasoning and language. Cipolotti et al. (1991) thus described the case of a lady, CG, who, after a left parietal lobe damage, was still proficient in language tasks and reasoning, but could not deal at all, verbally or otherwise, with numbers above four.

Specific error patterns and relative weaknesses may be a signature of specific neurological pathologies (see Cappelletti et al., 2012; Palmieri et al., 2013, for reviews). For instance, Delazer et al. (2006) found that in Posterior Cortical Atrophy, number comparison, approximation and number transcoding were severely impaired, but multiplication, addition facts and rules were preserved; and Palmieri et al. (2013) recently found that in Amyotrophic Lateral Sclerosis the largest majority of errors were in multiplication tasks.

It seems likely that the different specific deficits could impact in different ways on daily living, though this has up till now not been investigated. The instrument we are developing here can provide the foundation for such an investigation.

In one specific practical area, financial competence, there have been attempts to assess the impact of cognitive difficulties more generally on everyday life. Marson and co-workers provided a theoretical framework and appropriate tools to assess reduced financial competence in the elderly and in pathological conditions such as Alzheimer's disease, MCI or traumatic brain injury (Marson et al., 2000, 2009; Dreer et al., 2012; Martin et al., 2012). Studies by Webber and colleagues have focussed on the legal aspect of numerical competence, that is, whether an individual requires an administrator to manage some or all of his or her financial affairs, and to this end have developed the Financial Competence Assessment Inventory (FCAI) (Webber et al., 2002). For people with cognitive impairment (classified as acquired brain injury, dementia or psychiatric disorders), a positive correlation was found between the Arithmetic score on the WAIS-III and FCAI, an assessment scale of financial competence (Kershaw and Webber, 2008).

However, although these studies answered some important questions, they did not directly address the issue of how a specific deficit relates to the range of tasks that a patient can or cannot do in his or her everyday life. To take one example, Patient CG was a competent bookkeeper and manager of the family hotel, but as a consequence of a focal brain lesion, suffered very severe global acalculia, despite her otherwise spared cognitive skills, and therefore could no longer maintain her previous occupation. Patient BE (Hittmair-Delazer et al., 1994), an accountant, and patients ZA and TL (Girelli et al., 1996), all relatively young people, could go back effectively to the pre-morbid occupation or studies only when they were specifically treated for their very selective arithmetical facts retrieval deficits. The main aim of this study is to build and validate an instrument, the numerical activities of daily living (NADL), designed to differentiate different types of acquired acalculia, as a precondition for assessing the effects of these deficits on everyday life. We then sought to address the following questions:

- To what extent do these specific impairments relate to the difficulties measured by available scales evaluating activities of daily living in general (e.g., Instrumental Activities of Daily Living, IADL, Lawton and Brody, 1969; Katz, 1983)?
- To what extent does general deterioration of cognitive functions as measured by standard clinical instruments like the MMSE capture numerical deficits?

## Methods

Four new instruments were specifically designed for this investigation, and were administered to participants along with known clinical batteries.

### The NADL battery

The way NADL is structured allowed us to collect information about the degree of awareness of the deficit by the patient and by her or his caregivers.

NADL is divided into four parts (for details, see Appendix A, supplementary material).

1. The Patient Interview (Since this study involves a control group, the Patient Interview is referred to as the Participant Interview).
2. The Caregiver Interview.
3. The Informal Test, which is designed to offer a brief clinical assessment to determine whether the Formal Test of numerical abilities needs to be administered.
4. The Formal Test. This is a detailed assessment of the numerical abilities critical to daily living; these abilities are typically assessed in neuropsychological investigation of numerical and mathematical impairments. Thus, this test may be considered as an external criterion for the other subtests. In clinical practice, this only needs to be administered if there is evidence in the first three parts indicating a deficit in numerical abilities. However, for this study, it was always administered in order to evaluate the validity of the first three parts.

### Parts 1 and 2: participant interview and caregiver interview

These brief interviews, administered separately to the patient and to the caregiver, are meant to provide a rough assessment of the patient's awareness about his or her numerical deficit. The comparison of the patient's answers with those of the caregiver is designed to provide such information. These interviews consist of 10 simple questions (e.g., “Do you shop by yourself?”; “Do you make your own telephone calls unaided (i.e. do you dial them yourself)” on how well the participant uses numbers in everyday life, with equivalent questions asked of the participant and, in reference to the participant, of the caregiver. The activities were selected as relevant activities of daily living that are likely to be influenced by numerical abilities on the basis of previous literature and of clinical experience (frequent complaints about what patients cannot do any more and similar information).

### Part 3: the informal test of numerical competence

This test is meant to assess the numerical competence likely to be necessary in everyday life. It encompasses questions in the domains of Time (current date?), Measure (amount of pasta or rice in an average portion?), Transportation (distance between home and hospital?), Communication (own telephone number?), General Knowledge (days in a week?) and Money (cost of a car?). When the question implied an estimate rather than a precise number, the answers was considered correct if within a reasonable quantity interval (e.g., amount of pasta/rice: 80 g ±50 per person), The choice of these domains was made in consideration of previous literature and available instruments (e.g., Katz et al., 1963; Katz, 1983; Lawton and Brody, 1969). These domains (mostly chosen on the basis of most frequent patients' own complaints and those of their relatives) do not, of course, exhaust the range of tasks that might involve numerical abilities in the life of the participant. However, they can indicate potential difficulties in everyday life justifying further clinical assessment of mathematical functions.

### Part 4: the formal test of numerical abilities

This battery has been designed to assess the numerical abilities of patients using brief graded-difficulty subtests. The battery is organized in four sections consistent with previous neuropsychological batteries for numerical abilities (Delazer et al., 2003):

#### Section 1: number comprehension

This section comprises three subtests that test the patient's ability to relate number words and digits to numerical magnitudes: Numerosity Comparison (Comparing the number of squares in two panels presented simultaneously, up to nine squares per panel), Number Line marking (The participant is asked to mark a number on a line defined by its end points), and Digit Comprehension (10 panels, similar to the above, are presented one at a time along with a list of digits 1 to 10. For each panel, the participant points to the appropriate number).

#### Section 2: reading and writing arabic numerals

The aim of this section is to assess the ability to transcode between written and spoken numbers. This section is separate from that on calculation, since a dissociation has been observed in individual patients (Cipolotti and van Harskamp, 2001). The section consists of two subtests: Reading Numbers Aloud (including two digits, e.g. 12, up to five digits, e.g., 65300, numbers), and Writing Numbers on Dictation (including two digits up to five digits numbers).

#### Section 3: mental calculation

The aim of this section is to assess the ability to perform simple mental calculation (on spoken presentation). This section consists of three subtests: Mental Addition (e.g., 5 + 7), Mental Subtraction (e.g., 13 − 4), and Mental Multiplication (e.g., 9 × 6).

#### Section 4: written calculation

This section assesses in detail the components of calculation. The section consists of six subtests grouped in two subsections. The first subsection, named Understanding Arithmetical Rules and Principles, assesses the ability to use basic rules (e.g., operations with 0) and principles (e.g., the commutativity of addition) in written form. It consists of subtests for Arithmetical Rules (e.g., 0 + 9; 1 × 7), Addition Principles (e.g., 26 + 37 = 63 → 37 + 26 = ?), and Multiplication Principles (94 × 5 = 470 → 93 × 5= ?). The second subsection assesses the ability to perform more complex operations in writing. This latter subsection, named Written Operations consists of three subtests: Addition (e.g., 463 + 659), Subtraction (548 − 231), Multiplication (429 × 53).

### Mini mental scale examination (MMSE)

The MMSE (Mini-Mental-State Examination, Folstein et al., 1975) is the most widely used instrument to quickly evaluate the extent of general mental deterioration.

### The montreal cognitive assessment (MoCA)

The MoCA (Montreal Cognitive Assessment, Nasreddine et al., 2005) is a brief screening tool, more recent and slightly longer and more sensitive to Mild Cognitive Impairment (MCI) than MMSE. For this reason it was added to the most widely used MMSE.

### Instrumental activities of daily living (IADL)

IADL (Lawton and Brody, 1969; Katz, 1983) is a widely used scale, originally built in the attempt to assess everyday functional competence, that taps a level of functioning not captured by the more commonly used Activities of Daily Living scale (Katz et al., 1963). This scale collects information from the patient's caregiver about a series of functions concerned with a person's ability to cope with her/his environment in terms of familiar tasks: Use of the telephone; Shopping; Food Preparation; House Keeping; Laundry; Mode of Transportation; Responsibility for own Medication; Ability to Handle Finances. Importantly, some of these activities entail the use of numbers and calculation.

### Participants

A total of 323 volunteer participants took part in the study: a control group (*n* = 148) and a patient group (*n* = 175).

Participants of the control group were recruited in Italy. They were autonomous in their activities of daily living and, at the time of the assessment, they had no pathologies that could have influenced their cognitive status or its assessment. They had no record of developmental learning disorders. This group had a mean age of 53.05 years (*SD* = 16.80, range = 21–94), a mean education of 11.16 years (*SD* = 4.47, range = 5–26). Eighty-one were female.

Data from the control group were used to obtain normative cut-offs and some data from the control group were also included in the analysis assessing the psychometric properties of the battery.

Participants in the patient group were mostly recruited at the I.R.C.S.S. San Camillo on the Venice Lido or in the Policlinico of Padova. They gave their informed consent according to the Helsinki Declaration. The diagnosis of these patients was established through the standard protocols for their pathologies. The patient group had a mean age of 58 years (*SD* = 18.01, range = 18–90) and a mean education of 10.371 years (*SD* = 4.35, range = 2–19). Eighty-nine were female.

Details on demographic variables and neurological diagnosis of the patient group are reported in Table 1. Data from several different groups of patients were included for two reasons. First, to obtain variance in test scores in order to meaningfully assess the properties of the batteries (the control groups was expected to score at ceiling in most of the tests); second, to allow a preliminary investigation comparing specific profiles in numerical and math deficits in different neurological diseases.

**Table 1:** **Demographic variables of the patient group**.

**Diagnosis**	**Age mean (*SD*)**	**Education mean (*SD*)**	**Gender as number of female (percentage of female)**	***N***
Alzheimer	74.07 (7.81)	8.69 (4.85)	8 (61%)	13
Mild cognitive impairment	75.08 (7.81)	8.75 (3.85)	15 (42%)	36
Parkinson	64.82 (9.07)	10.75 (4.91)	13 (46%)	28
Multiple sclerosis	51.28 (13.25)	10.67 (3.87)	32 (53%)	60
Neurofibromatosis type 1	28.40 (9.18)	12.64 (3.98)	13 (59%)	22
Corticobasal degeneration	73 (-)	5 (-)	1 (50%)	1
Frontotemporal dementia	65 (-)	13 (-)	0 (0%)	1
Lewy body dementia	76 (-)	17 (-)	0 (0%)	1
Left hemisphere lesion	56 (13.07)	11 (4.86)	5 (71%)	7
Right hemisphere lesion	67 (1.71)	11 (5.31)	1 (25%)	4
Amyotrophic lateral sclerosis	65 (2.83)	5 (-)	1 (50%)	2
Overall patients	58.23 (18.01)	10.37 (4.35)	89 (51%)	175

*Age and education are reported as number of years. The Gender column indicates the number of female participants. N indicates the number of participants in the group*.

### Procedure

All participants were tested individually. Testing was administered by trained neuropsychologists, always in the following order: first the *Participant Interview*, followed by the *Informal test* and then by the *Formal test*. The *Caregiver Interview* was administered to a caregiver or to a close relative of the patient, in the patient group, at a separate time. Additionally, the *Caregiver Interview* was also administered to a relative of normal controls, even though it was expected that almost all of these reports would be at ceiling.

Together with the NADL battery, all participants were administered with MMSE and MoCA. The caregiver was also asked to compile the IADL (Instrumental Activities of Daily Living, Katz, 1983).

All statistical analyses were performed with the free statistical software *R* (R Core Team, 2012).

#### Psychometric properties

Several psychometric properties of NADL were investigated. To limit the extent of ceiling effect, this analysis was performed only on patients.

*Internal consistency* was calculated by means of standardized Cronbach's alpha. The consistency of the whole *formal test* was satisfactory, with a Cronbach's alpha of 0.73. The consistency of each subtest was also evaluated. The average of Cronbach's alpha was 0.59. *Numerosity comparison* and *number line* showed the highest Cronbach's alpha (0.8 and 0.78, respectively), while *writing number to dictation* and *reading number aloud* showed the lowest Cronbach's alpha (0.37 and 0.19, respectively). All results are reported in Table 2. The very low scores on some subtests are not surprising, since in these tests even patients' performance was almost at ceiling. However, we decided to keep these tests because NADL is designed for patients with neuropsychological disorders: some patients may therefore show specific impairments and variability in those subtests, even if our sample of neurological patients performed at ceiling. Notably, low consistency was observed only in tests showing performance almost at ceiling.

**Table 2:** **Reliability of NADL**.

	**Cronbach's alpha**	**Test–retest reliability**	**Inter-rater reliability**
Interview with patients	0.5	0.69	0.82
Interview with caregiver	0.62	0.98	1
Informal total	0.64	0.86	0.94
Formal total	0.73	0.86	0.87
Numerosity comparison	0.8	0.51	0.84
Number line	0.73	0.50	0.60
Digit comparison	0.71	0.32	0.9
Reading number aloud	0.37	0.20	0.68
Writing numbers on dictation	0.19	0.24	0.2
Mental addition	0.7	0.28	0.58
Mental subtraction	0.5	0.44	0.95
Mental multiplication	0.61	0.72	0.92
Written rules	0.59	0.38	0.55
Written addition	0.65	0.68	0.81
Written multiplication	0.63	0.46	0.62
Written operations—addition	0.6	0.60	0.66
Written operations—subtraction	0.71	0.50	0.83
Written operations—multiplication	0.6	0.38	0.55
Total number comprehension	0.43	0.34	0.78
Total reading and writing Arabic numerals	0.39	0.39	0.62
Total mental calculation	0.58	0.76	0.93
Total rules and principles	0.78	0.88	0.92
Total written calculation	0.74	0.47	0.68

*The Table reports reliability scores for NADL*.

*Test–retest reliability* was assessed in a subsample of 19 participants, from the sample of patients with a neurological disease. All participants were tested within a month interval between the two observations. A Spearman correlation was utilized as an index of test–retest reliability. The test–retest reliability of the single subtests ranged from 0.20 to 0.98. As in the case of Cronbach's alpha, the low values in some subtests are a consequence of the scores almost at ceiling observed in the sample considered. These results, rather than indicating a low reliability, indicate that the large majority of participants performed at ceiling in both test and retest; the few participants that departed affected the results, given the relatively small score range of the subtests. These data should be taken into account when using those tests in the assessment of change in the ability of the patient due to recovery, intervention or both.

The *Inter-rater reliability* of NADL was assessed by means of Intra-Class correlations (ICC) on a subset of 14 patients. Two examiners separately scored their performance. The ICC was very high in almost every test, supporting a high objectivity (that is, independence from subjective judgments in attributing the scores) of the overall battery.

#### Cut-offs

Cut-off scores based on the distribution of scores in the healthy participant group were calculated. Cut-offs were calculated separately for each subtest of the formal test and for the informal test, but also for each section of formal and informal test, and for the global scores of formal and informal tests. To account for the effect of demographic variables (age, education, and gender) on cut-offs we used the results of regressions with the subtest or test scores as dependent variables and the demographic variables as predictors (the analysis are reported in detail in the paragraph *age and education effects*). The residuals of the regression models built can be conceived as *adjusted scores*, i.e., what remains of the observed scores after the effects of demographic variables are removed. Cut-off were calculated as 5th percentile of adjusted scores in the control sample, for those tests in which a significant effect of demographic variable was found. The advantage of a regression method approach is that it allows using the whole normative sample to have a single cut-off for each score (and not cut-offs stratified for age, education and gender). Before comparing an observed subtest or test score with a cut-off, the effect of demographic variables is removed (if significant), utilizing the same regression model that was used to obtain the cut-offs, with the following procedure: first a predicted score of the participant is calculated by entering his/her values for the relevant demographic variables (age, education, and gender) in the regression model obtained on the control group sample (Table 3); then, the predicted score is subtracted from the observed score to obtain the residual, that is the *adjusted score* for that given participant. If a demographic variable shows no effect on a score, than there is no reason to take into account that variable in calculating the cut-offs. In such case the cut-offs were calculated as 5th percentile of the raw scores. An spreadsheet reporting cut-offs is provided in supplementary material. This file, once entered with a patient's data, automatically shows if the score is below cut-off in each subtest.

**Table 3:** **Regression models for cut-off corrections of NADL global scores and scores on subtests**.

	**Intercept (*SE*)**	**Age (*SE*)**	**Education (*SE*)**	**Gender (*SE*)**	***R*^2^**
Interview with patients	10.57 (0.21)	−0.02 (0.004)	–	–	–
Interview with caregiver	–	–	–	–	–
Informal total	23.38 (0.49)	−0.05 (0.008)	–	–	0.18
Formal total	70.78 (2.45)	−0.10 (0.03)	0.39 (0.11)	–	0.26
Numerosity comparison	–	–	–	–	–
Number line					
Digit comprehension	–	–	–	–	–
Reading number aloud	4.08 (0.13)	–	0.04 (0.01)	0.20 (0.09)	0.11
Writing numbers on dictation	–	–	–	–	–
Mental addition	–	–	–	–	–
Mental subtraction	–	–	–	–	–
Mental multiplication	4.45 (0.22)	–	0.07 (0.02)	–	0.09
Written rules	6.27 (0.38)	−0.02 (0.004)	0.07 (0.02)	–	0.30
Written addition	3.42 (0.44)	−0.02 (0.005)	0.07 (0.02)	–	0.25
Written multiplication	4.66 (0.21)	−0.02 (0.004)	–	–	0.21
Written operations—addition	–	–	–	–	–
Written operations—subtraction	6.49 (0.27)	−0.02 (0.004)	–	–	0.13
Written operations—multiplication	–	–	–	–	–
Total number comprehension	–	–	–	–	–
Total reading and writing Arabic numerals	9.54 (0.17)	–	0.06 (0.01)	0.25 (0.12)	0.14
Total mental calculation	15.96 (0.35)	–	0.09 (0.03)	–	0.05
Total rules and principles	13.74 (0.85)	−0.05 (0.01)	0.17 (0.04)	–	0.40
Total written operations	17.02 (0.54)	−0.04 (0.01)	–	–	0.11

*Each row in the table reports the coefficients of one linear regression model. The first column reports the dependent variable, the following four columns report the parameter for the models (the value within brackets indicates the standard error for the parameter). The last column reports the adjusted R-squared. A missing value in the table indicates that the parameter did not contribute significantly to the model fit and then was removed in the modeling procedure. All coefficients reported (with the exception of Intercept, which is always included in a meaningful model) were selected by mean of backward elimination of non-significant variables. All variables reported significantly improved the fit of the model and their associated t-values had p < 0.05*.

#### Construct validity of the test

Since NADL is the first battery with the aim of assessing the impact of numerical deficits in daily living, we cannot use external evidence of actual daily living to assess its validity. However, it is possible to collect evidence on the construct validity of the test by investigating correlations among test sections and among the tests and other external tests. Thus, we inspected the correlation among the parts of NADL and with the other tests administered to investigate if the results support the claim that the parts of NADL are indeed measuring the construct of numerical activities related to numbers. Importantly, the formal test of NADL can be considered as an external criterion for the other parts, since it covers the main domains of neuropsychological models of mathematical and numerical abilities, and it closely resembles other existing batteries (see for example, Delazer et al., 2003).

The results of correlations on NADL global scores and scores on subtests are reported in Table 4. Since data from the Caregiver Interview were not available for all the participants, the data of these correlations come from a subset of 141 participants for whom this data was available, evenly distributed among control participants and neurological patients. All statistical analyses reported in this paragraph included this sample of healthy controls and patients.

**Table 4:** **Correlation between NADL and other tests**.

	**Interview with patient**	**Interview with caregiver**	**Informal total**	**Formal total**	**MMSE**	**MoCA**
Interview with caregiver	0.82					
Informal total	0.47	0.58				
Formal total	0.54	0.64	0.65			
MMSE	0.60	0.74	0.65	0.75		
MoCA	0.46	0.61	0.51	0.58	0.74	
IADL	0.73	0.81	0.51	0.57	0.65	0.54

*All correlations showed in Table 4 are significant at p < 0.001 (n = 141). The participants included in these correlations were both healthy controls and patients with neurological diseases*.

The overall pattern of correlations suggests a source of communality that may underlie the interrelation among scores on the different tests. This is not surprising, because all of the tests are supposed to be influenced by the overall cognitive status of the individual.

These results thus support the construct validity of NADL and suggest that the interview with the caregiver is a better estimate of numerical abilities than the interview with the patient.

#### Awareness of numerical deficits in neurological patients

The interviews with the participant and with the caregiver give the opportunity to estimate the numerical competence in the life of the patients. Results of the correlations suggest that the estimate of the caregiver on the impact of numerical deficits in daily living is better then the estimate made by the patients themselves (see previous paragraph). The error bias in patients was further explored by comparing the scores on the interviews by means of paired *t*-tests. For this analysis control subjects and patients were analyzed separately, since control subjects mostly scored at ceiling in the interviews. The analysis on control subjects showed no difference between the interview with the participant and the interview with their relative [*t* = 0.469, *df* = 77, *p* = 0.64]. The analysis of patients showed a significant difference, with higher scores in the patient interview compared with the caregiver interview [*t* = 4.41, *df* = 62, *p* < 0.001]. In summary, the patients tend to overestimate their abilities as compared to the judgment made by the caregivers.

## Discussion

The main purpose of this study was to obtain an instrument able to assess numerical activities of daily living. The instrument described here, the NADL battery, has been normed on a sufficient number of participants, including control participants varying in the age, and its psychometric properties have been tested on participants affected by a wide variety of pathological conditions.

We have found that the NADL battery shows a good reliability both in terms of test-retest and inter-rater reliability. The internal consistency was satisfactory as well. Construct validity was also satisfactory, as tested by the correlation between NADL parts and cognitive status as assessed by MoCA and MMSE, and with IADL. Importantly, the Patient Interview and Caregiver Interview correlated well with IADL, and with the Informal and Formal parts. The Caregiver Interview was a better predictor of actual numerical ability, with additional finding that the Patient Interview overestimated numerical abilities. This confirms the NADL strategy of using these interviews as a brief clinical screening tool to see whether a more detailed investigation of the patient's numerical abilities is indicated.

Since patients tended to overestimate their own numerical competence, a specialized numerical battery, such as NADL, can be employed to evaluate numerical competence for financial or legal decisions about the ability of an individual to manage his or her own affairs, especially financial affairs. The need of such evaluation is likely to increase with the elderly, irrespective of clear neurological damage (Webber et al., 2002).

It is known that distinct patterns of numerical deficits have been identified for a number of neurological conditions, including progressive diseases, such as Alzheimer's disease, Temporal Lobe Epilepsy, Fronto-temporal Dementia, Semantic Dementia and other forms of Primary Progressive Aphasia, Amyotrophic Lateral Sclerosis, Posterior Cortical Atrophy, Parkinson's Disease and other diseases of basal ganglia (see Palmieri et al., 2013 for a review) and genetic defects (e.g., Prader Willi Syndrome, Semenza et al., 2008; X Fragile Premutation, Semenza et al., 2012; Turner Syndrome, Bruandet et al., 2004). These studies have been conducted with different assessment tools and with patients with differential severity of numerical deficit. The pathological profile obtained in these studies depends on a combination of the effect on damage on specific neural networks, and also on the type of test that has been used and by the overall degree of cognitive deficit. It is thus hard to compare these studies with each other, because the outcome of each individual study might heavily depend on type of test, degree of severity and other factors like, in particular, age and education that may vary widely. In future, the standardized use of battery such as NADL will enable clinicians and researchers to compare the numerical abilities and disabilities in different conditions more systematically.

In our study, we were able to compare for the first time on the same battery Parkinson's Disease and Multiple Sclerosis. On Total Written Calculation, patients with Parkinson performed worse than patients with Multiple Sclerosis, irrespective of the severity of the condition, general cognitive impairment, age or education. Similarly, neurofibromatosis, a pathological condition for which no previous investigation of numerical abilities was available, and where the pathology shows much earlier than in the above-mentioned degenerative diseases, is characterized by a distinct profile of numerical deficits, especially mental calculation, again independently from severity of the disease, age, and education.

NADL may also be useful in the assessment of “cognitive or brain reserve” the resilience of function in the context of neural damage (see, for example, Nucci et al., 2012). It has been widely claimed that high educational level acts as a protective factor, and here we can test the more specific prediction of whether a high level of mathematical education level protects against slowing or deterioration of mathematical abilities with aging and disease.

The NADL battery is an initial step in developing an efficient assessment tool. A shorter version of the Formal part may be needed. However, the detailed and extensive investigation presented here provides the basis on which to proceed. Subsequent analysis of the data from patients and controls may eventually enable us to include only the most discriminating items, and thereby reduce the length of the battery. Importantly, however, the interview with the caregiver seems to provide a first, rough but quite reliable, estimate of a patient's numerical skills. It may thus be used when a thorough evaluation cannot be done, or to guide a screening decision.

The present battery enables the investigation of the consequences of a given numerical defect on numerical activities of daily living. This is work in progress. Factors such as the influence of relative severity will be the focus of subsequent studies. At the present stage, however, this battery can already be employed in its present form to assess patients for their rehabilitation or retraining and to monitor and assess the outcome of rehabilitation and retraining in a real life setting.

### Conflict of interest statement

The authors declare that the research was conducted in the absence of any commercial or financial relationships that could be construed as a potential conflict of interest.
