# Supplementary material for: A new clinical tool for assessing numerical abilities in neurological diseases: numerical activities of daily living
Source: Front Aging Neurosci. 2014 Jun 20;6:112. doi: 10.3389/fnagi.2014.00112 (PMC4115360; doi:10.3389/fnagi.2014.00112)
Supplement: Supplementary file 1 [file DataSheet1.DOC]

# NUMERICAL ACTIVITIES

# OF DAILY LIVING

# (NADL)

# by

# Carlo Semenza

# I.R.C.C.S. Ospedale San Camillo, Lido, Venezia;

# Dipartimento di Neuroscienze, Università di Padova

# Francesca Meneghello

# I.R.C.C.S. Ospedale San Camillo, Lido, Venezia;

# Brian Butterworth

# I.R.C.C.S. Ospedale San Camillo, Lido, Venezia;

# Institute of Cognitive Neuroscience, UCL

# Introduction

This battery has three purposes:

1. To assess the numerical abilities of patients using brief graded difficulty tests.
2. To provide the basis for subsequent intervention based on specific patterns of deficit.
3. To better interpret the results of the Numerical Activities of Daily Living; that is, to evaluate how activities of daily living depend on specific numerical abilities.

The battery is organized in three levels:

The first level assesses the patient’s ability to comprehend numbers. If the patient fails these comprehension tests, then it may be worth continuing only with transcoding tests.

The second level for patients who pass the comprehension tests, is to assess separately transcoding and calculation, since a dissociation has been observed in individual patients.

The third level assesses the components of transcoding and calculation in detail, while the fourth level assesse types of error and subtypes within the third level.

From previous research, the nature of the errors, as well as the overall score, is an important diagnostic for identifying cognitive status of the patient. It is thus of the utmost importance to keep documented track of all the errors.

This battery is an initial step in developing an efficient assessment tool. Although this may seem to be a very long battery to give to patients, it is crucial at this stage to have the complete battery administered and scored. Subsequent analysis of the data from patients and controls will enable us to include only the most discriminating items, and thereby reduce the length of the battery.

# Number comprehension

## Numerosity comparison.

### Comparing the number of squares in two panels presented simultaneously (up to nine squares per panel) with area orthogonal.

**Numerical difference**. There are three levels of differences in the number of squares in each panel 3, 2 and 1

**Congruency conditions**. There are three levels of congruence. In the congruent condition, the panel with most squares also has the greatest area covered by the squares. In the neutral condition, the areas covered by the squares is the same in both panels. In the incongruent condition, the panel with the most squares has less area covered.

There is a total of 6 items: 2 levels of congruency x 3 levels of difference.

There is one practice item.

### Instructions.

“Point to the panel with the most squares.”

(Counting is allowed).

### Scoring

One point for each correct decision. (Maximum = 6)

## 2. Number line task:

The participant is asked to mark a number on a line defined by its end points. There are separate lines for each trial. There are three conditions of increasing difficulty defined by the end points.

The line is always twenty centimetres.

Condition 1 line 0-10. Mark 5, 2 and 7;

Condition 2 line 0-100. Mark 50, 25 and 75;

Condition 3 line 0-1000. Mark 250, 750 and 125.

One practice item in Condition 1. Mark 9.

### Scoring

Use ruler to record deviation plus or minus in mm. The score is absolute value of the deviation (disregarding the sign). A mean deviation per condition of 5mm or less is scored as 1 point.

## 3. Digit comprehension.

Ten panels, similar to the above, are presented one at a time along with a list of digits 1 to 10. For each panel, the participant points to the appropriate number. One practice item.

### Instructions.

“Point to the number corresponding to the number of squares.”

(Counting is allowed).

### Scoring

One point for each correct decision. (Maximum = 10).

## Calculate total number comprehension.

**B. Reading and writing numbers**

## 4. Reading Arabic numerals (4); 12; 53; 104; 2600; 65300

## 5. Writing Arabic numerals to dictation (4); 2; 51; 307; 2005; 42300

Score errors: syntactic vs lexical

(For example if “two hundred and twelve” is written, 20012, that is a syntactic error; if it is written 312, that is a lexical error. However, it is important to record each for subsequent analysis. )

## B. Calculate reading and writing numbers total

# C. Mental calculation

Here mental calculation is tested. There are three levels presented in the following order:

## 6. Mental calculation

Addition level. 2+3; 5+2; 2+1; 7+7; 5+7; 9+6

Subtraction level. 2-1; 9-6; 6-2; 16-8; 13-4; 11-4

Multiplication level. 8x2; 5x6; 3x9; 8x7; 9x6; 8x9

There is one practice item per level.

### Instructions

“Please tell me the answer to this problem.” (Repeat this before each problem, unless the participant fully understands the task, in which case, just present the problem in the following form:

“What is one plus six?”

“What is seven minus three?”

“What is five times three?” OR “What is five multiplied by three?” depending on what is easier for the participant.

(The participant may ask for one repetition, but is not allowed to write down anything.)

Addition practice item: 1+6

Subtraction practice item: 7-3

Multiplication practice item: 5x3

### Scoring

Score one point for each correct answer. Maximum 6 for each level.

## C. Calculate mental calculation total

# Written calculation

## 7. Understanding arithmetical rules and principles

## Instructions

Here it is important to explain that these problems can be solved without calculation.

## Scoring

Score 1 point for each correct answer.

**Rules**

Addition rules 0+9=?; 7+0=?

Subtraction rules 7-0=?; 3-3=?

Multiplication rules 0x7=?; 1x6=?; 4x0 =?

Maximum = 7

## Addition Principles

### Instructions

Demonstrate that 42+19=61 🡪 19+42=? can be solved without calculating 42+19=61. The participant should be encouraged to think through each problem rather than to begin calculating.

24+37=61 🡪 37+24=?

68+43=111 🡪 111-43=?

37+18=55 🡪 370+180=?

60+29=89 🡪61+29=?

Maximum = 4

## Multiplication Principles

Demonstrate that 22x31=682🡪31x22=? can be solved without calculating 31x22. The participant should be encouraged to think through each problem rather than to begin calculating.

56x17=952🡪17X56=?

34x6=204🡪204÷6=?

64x5=320🡪64+64+64+64+64=?

94X5=470🡪93x5=?

Maximum = 4

**8. Written operations**

Here participants are asked to calculate the answer to questions written on the question sheet.

### Instructions

Explain the task saying, “There is no time limit, but if you are stuck on one question move on to the next.”

If the participant has difficulty writing but not speaking, offer to write down the answers to dictation.

There are three levels of operation: addition, subtraction and multiplication; and two levels of difficulty within each: addition no carrying, addition carrying; subtraction no borrowing, subtraction borrowing; multiplication no carrying, multiplication carrying.

### Scoring

Score one point for each correct answer. Maximum 6 for each of addition and subtraction; maximum of 5 for multiplication.

**Addition**

No carrying: 43+52=? 657+231=? 749+120=?

Carrying: 58+43=? 463+659=? 825+287=?

**Subtraction**

No borrowing: 79-34=? 548-231=? 456-132=?

Borrowing: 62-18=? 317-126=? 632-278=?

**Multiplication**

No carrying: 54x2=?

Carrying: 25x8=? 34x 16=? 429x53=? 618x203=?

# Participant details

Name

Date of birth

Education (years)

Medical History

Date of test

Patient number (if relevant)

Examiner: please read the instructions before starting the tests.

# Part 1

# Interview with patient or participant

# Interview with patient

| General |  |
| --- | --- |
| Do you get confused by numbers | 1 |
| Time |  |
| Are you typically punctual for appointments | 1 |
| Measure |  |
| Do you administer your own drugs | 1 |
| Money |  |
| Do you shop by yourself | 1 |
| Do you give the right money, get the right change | 2 |
| Do you check your bank account | 1 |
| Transportation |  |
| Can you take the correct train/bus unaided | 1 |
| Communication |  |
| Do you make your own telephone calls unaided (i.e. do you dial them yourself) | 1 |
| Can you select TV channels correctly | 1 |
|  | 10 |

# Part 2

# Interview with caregiver

# Interview with caregiver

| General |  |
| --- | --- |
| Does s/he get confused by numbers | 1 |
| Time |  |
| Is s/he typically punctual for appointments | 1 |
| Measure |  |
| Does s/he administer her/his own drugs | 1 |
| Money |  |
| Does s/he shop by yourself | 1 |
| Does s/he give the right money, get the right change | 2 |
| Does s/he check her/his bank account | 1 |
| Transportation |  |
| Can s/he take the correct train/bus unaided | 1 |
| Communication |  |
| Does s/he make her/his telephone calls unaided (i.e. does s/he dial them yourself) | 1 |
| Can s/he select TV channels correctly | 1 |
|  | 10 |

# Part 3

# Informal test of numerical competence

# Informal test of numerical competence

| Time | Exact/reasonable answer |
| --- | --- |
| Current time – (±15 min) | 1 |
| Current day, year - exact | 2 |
| How long have you waited? **or** How long are we speaking? (± 15 min) | 1 |
| How long till 12 (if morning)/ 5 (if afternoon) (within ± 15 mins.) | 1 |
| Measure |  |
|  |  |
| How much pasta/rice for each person (80 g ±50)- reasonable | 1 |
| Transportation |  |
| Distance in km between home and hospital - reasonable | 1 |
| Communication |  |
| What is your telephone number? - exact | 1 |
| General knowledge |  |
| Age - exact | 1 |
| Date of birth | 1 |
| House number - exact | 1 |
| Days of week/months in year - exact | 2 |
| Dates of last war/attack on twin towers - exact | 2 |
| Money |  |
| How much is a new car (€7000-100000) | 1 |
| How much is a house (€100000-2m) | 1 |
| If a shirt normally costs €50, but is reduced by 10% how much will you have to pay? – (45) exact | 1 |
| Coin usage |  |
| **Pay for newspaper (€1.2)** given €1 and €2. Correct=Give €2 | 1 |
| Check change (60c). Correct=Say incorrect | 1 |
| Say correct change (80c) or additional change (20c) | 1 |
| **Give correct money (€5.50)** given 2, 2, 2, 1, 1, 1, 50c, 50c, 50c. If 2,2,1, 50c | 2 |
| If correct in any other combination | 1 |
| MAX | 23 |

# Part 4

# Formal assessment

# 1. Numerosity comparison test

Practice item


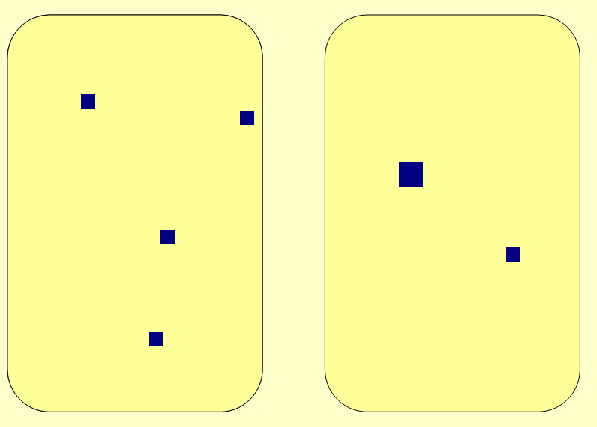


1


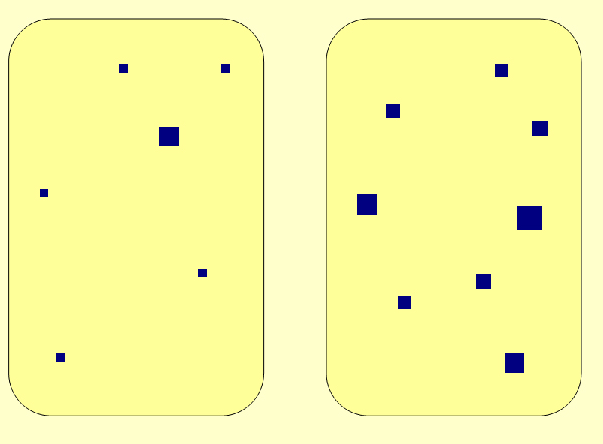


2


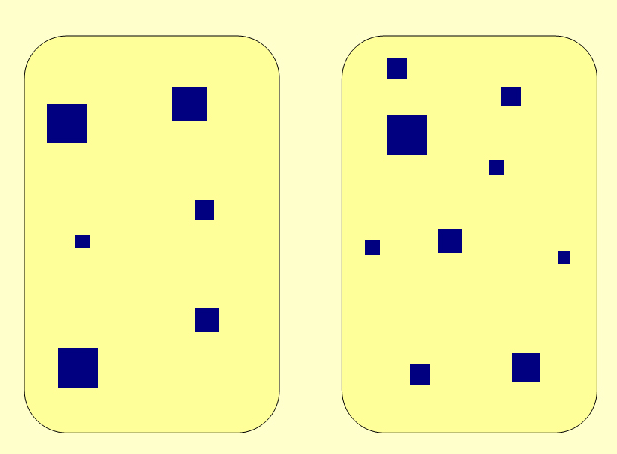


3


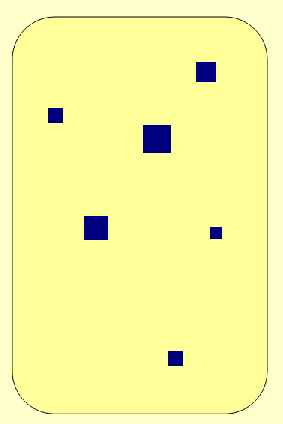

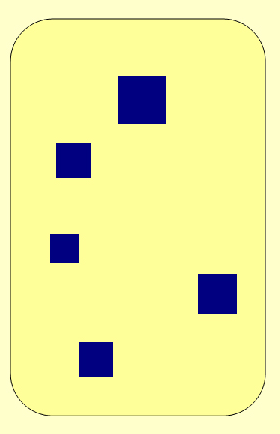


4


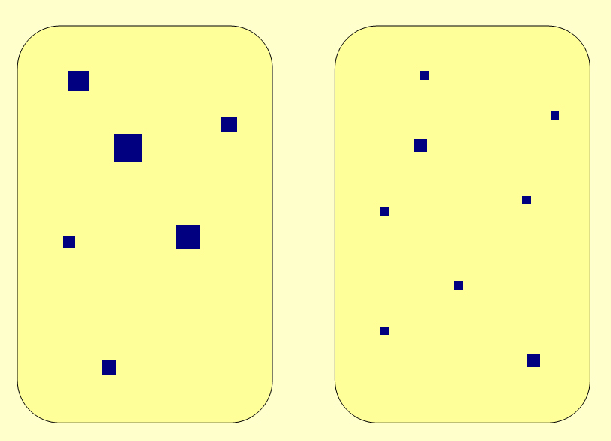


5


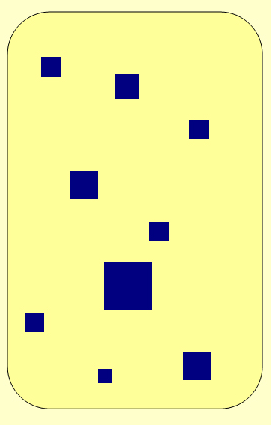

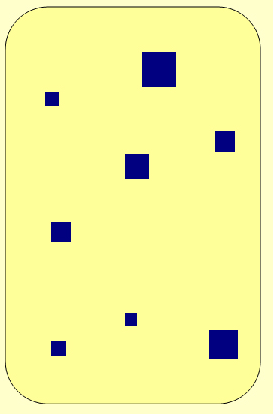


6


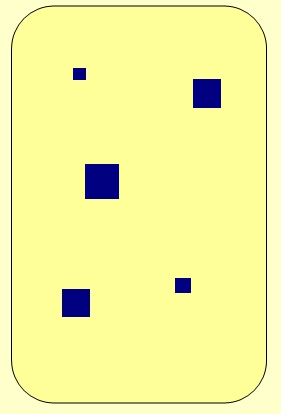

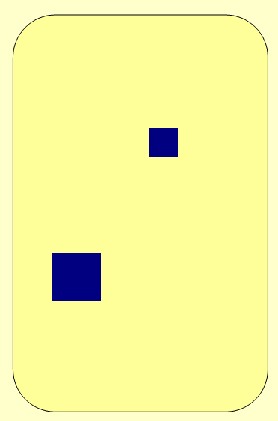


# 2. Number line test

PRACTICE

**3**

**0 10**

**5**

**0 10**

**2**

**0 10**

**7**

**0 10**

**50**

**0 100**

**25**

**0 100**

**75**

**0 100**

**250**

**0 1000**

**750**

**0 1000**

**125**

**0 1000**

# 3. Digit comprehension test

Point to the digit corresponding to the number of squares

**PRACTICE ITEM**


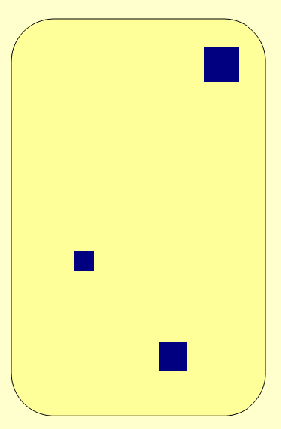


**1 2 3 4 5 6 7 8 9 10**


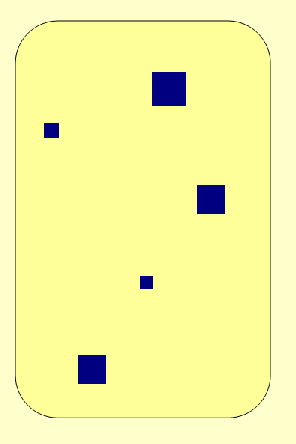


**1 2 3 4 5 6 7 8 9 10**

**
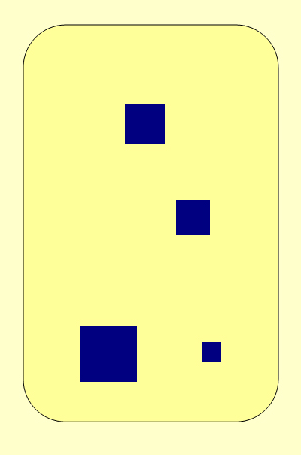
**

1. **2 3 4 5 6 7 8 9 10**


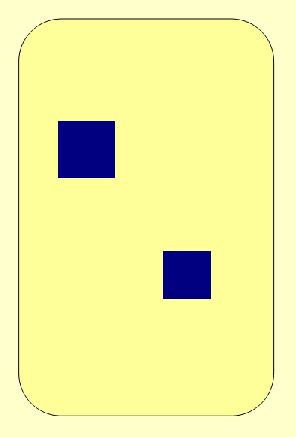


**1 2 3 4 5 6 7 8 9 10**


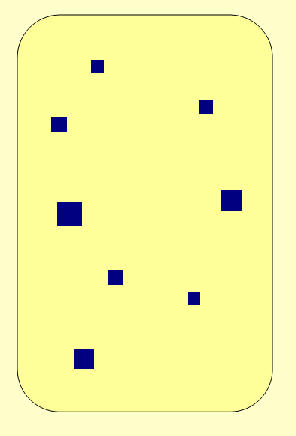


**1 2 3 4 5 6 7 8 9 10**


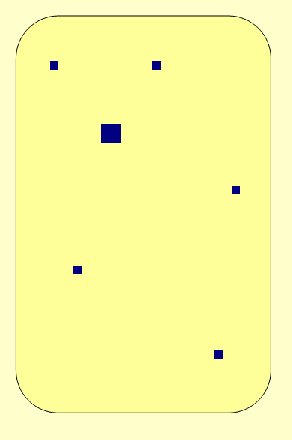


**1 2 3 4 5 6 7 8 9 10**


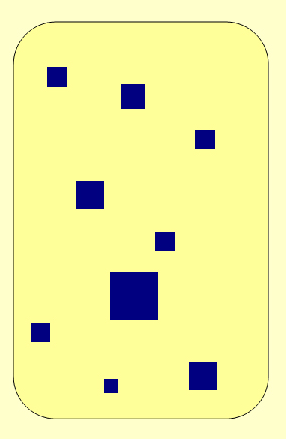


**1 2 3 4 5 6 7 8 9 10**


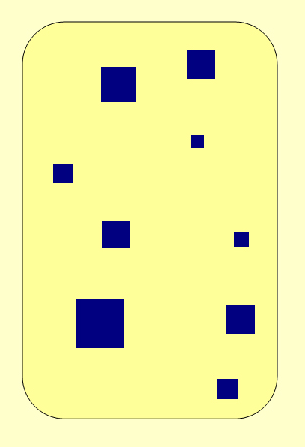


**1 2 3 4 5 6 7 8 9 10**


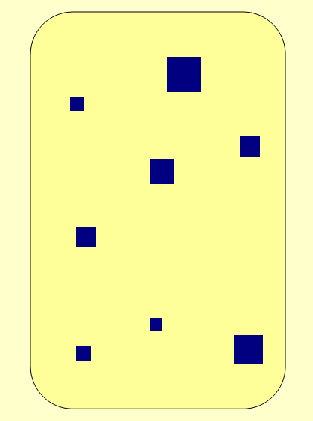


**1 2 3 4 5 6 7 8 9 10**


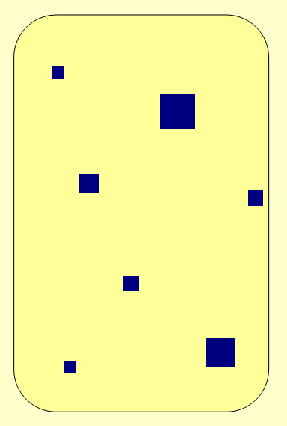


**1 2 3 4 5 6 7 8 9 10**


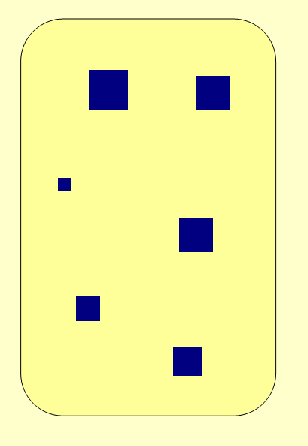


**1 2 3 4 5 6 7 8 9 10**

# 4. Reading numbers aloud

Practice item

4

12

53

104

2600

65300

# 5. Writing numbers to dictation

(see score sheet for numbers to dictate)

# 6. Mental calculation

(Oral presentation. See score sheet for questions to present)

# 7. Written calculation

(see score sheet before beginning)

0+9=

7+0=

7-0=

3-3=

0x7=

1x6=

4x0=

Please solve these problems without calculating

Practice item

42+19 = 61 🡪 19+42 =

26+37 = 63 🡪 37+26 =

68+43 = 111 🡪 111-43 =

37+18 = 55 🡪 370+180 =

60+29 = 89 🡪 61+29 =

Practice item

22x31 = 682 🡪 31x22 =

56x17 = 952 🡪 17x56 =

34x6 = 204 🡪 204/6 =

64x5 = 320 🡪 64+64+64+64+64=

94x5 = 470 🡪 93x5=

**8. Written operations**

43+52=

657+231

749+120=

58+43=

463+659=

825+287=

79-34=

548-231=

456-132=

62-18=

317-126=

632-278=

54x2=

25x8=

34x16=

429x53=

618x203=

# PART 5

# Score sheet

# A. NUMBER COMPREHENSION

# 1. Numerosity comparison

Question: Which panel has more squares?

**Practice**

| Item | Congruency & difference | Answer | Score |
| --- | --- | --- | --- |
| 1 | C(ongruent) 2 | R |  |
| 2 | C 3 | R |  |
| 3 | I 1 | L |  |
| 4 | I 2 | R |  |
| 5 | C 1 | L |  |
| 6 | I 3 | L |  |
| **Total** | | | **/6** |

# 2. Number line task (deviations in mm)

**Practice 3**

| **0-10** |  |
| --- | --- |
| 5 |  |
| 2 |  |
| 7 |  |
| Total deviation in mm |  |
| **1-100** |  |
| 50 |  |
| 25 |  |
| 75 |  |
| Total deviation in mm |  |
| **0-1000** |  |
| 250 |  |
| 750 |  |
| 125 |  |
| Total deviation in mm |  |
| **Total** | **/3** |

# 3. Digit comprehension

| Digit | Score |
| --- | --- |
| 5 |  |
| 4 |  |
| 2 |  |
| 8 |  |
| 6 |  |
| 9 |  |
| 9 |  |
| 8 |  |
| 7 |  |
| 6 |  |
| **Total** | **/10** |

# A. Total number comprehension /19

# B. READING AND WRITING NUMBERS

# 4. Reading Arabic numerals

Practice 4

| Question | Score |
| --- | --- |
| 12 |  |
| 53 |  |
| 104 |  |
| 2600 |  |
| 65300 |  |
| **Total** | **/5** |

**Errors**

Lexical

Syntactic

# 5. Writing Arabic numerals to dictation

Practice 4

| Question | Score |
| --- | --- |
| 2 |  |
| 51 |  |
| 307 |  |
| 2005 |  |
| 42300 |  |
| **Total** | **/5** |

**Errors**

Lexical

Syntactic

# B. Total reading and writing numbers: /10

# C. MENTAL CALCULATION

# 6. Mental calculation–Oral presentation

| Question | Answer | Score |
| --- | --- | --- |
| Practice1+6 | 7 |  |
|  |  |  |
| 2+3 | 5 |  |
| 5+2 | 7 |  |
| 2+1 | 3 |  |
| 7+7 | 14 |  |
| 5+7 | 12 |  |
| 9+6 | 15 |  |
| **Addition total** | | **/6** |

| Question | Answer | Score |
| --- | --- | --- |
| Practice 7-3 | 4 |  |
|  |  |  |
| 2-1 | 1 |  |
| 9-6 | 3 |  |
| 6-2 | 4 |  |
| 16-8 | 8 |  |
| 13-4 | 9 |  |
| 11-4 | 7 |  |
| **Subtraction total** | | **/6** |

| Question | Answer | Score |
| --- | --- | --- |
| Practice 5x3 | 15 |  |
|  |  |  |
| 8x2 | 16 |  |
| 5x6 | 30 |  |
| 3x9 | 27 |  |
| 8x7 | 56 |  |
| 9x6 | 54 |  |
| 8x9 | 72 |  |
| **Multiplication Total** | | **/6** |

# C. Total mental calculation: /18

# D. WRITTEN CALCULATION

## 7. Understanding arithmetical rules and principles

**Rules**

| Question and answer | Answer | Score |
| --- | --- | --- |
| 0+9= | 9 |  |
| 7+0= | 7 |  |
|  |  |  |
| 7-0= | 7 |  |
| 3-3= | 0 |  |
|  |  |  |
| 0x7= | 0 |  |
| 1x6= | 6 |  |
| 4x0= | 0 |  |
| **Total rules** | | **/7** |

**Principles**

| Question | Answer | Score |
| --- | --- | --- |
| Practice 42+19 = 61 🡪 19+42 = | 61 |  |
|  |  |  |
| 26+37 = 63 🡪 37+26 = | 63 |  |
| 68+43 = 111 🡪 111-43 = | 68 |  |
| 37+18 = 55 🡪 370+180 = | 550 |  |
| 60+29 = 89 🡪 61+29 = | 90 |  |
| **Total addition** | | **/4** |

| Question | Answer | Score |
| --- | --- | --- |
| Practice 22x31 = 682 🡪 31x22 = | 682 |  |
|  |  |  |
| 56x17 = 952 🡪 17X56 = | 952 |  |
| 34x6 = 204 🡪 204÷6 = | 34 |  |
| 64x5 = 320 🡪 64+64+64+64+64= | 320 |  |
| 94x5 = 470 🡪 93x5= | 465 |  |
| **Total multiplication** | | **/4** |

# D. Total Written Calculation: /15

**8. Written operations**

**Written addition**

| Question | Answer | Score |
| --- | --- | --- |
| 43+52 | 95 |  |
| 657+231 | 888 |  |
| 749+120 | 869 |  |
|  |  |  |
| 58+43 | 101 |  |
| 463+659 | 1122 |  |
| 825+287 | 1112 |  |
| **Total addition** | | **/6** |

**Written subtraction**

| Question | Answer | Score |
| --- | --- | --- |
| 79-34 | 45 |  |
| 548-231 | 317 |  |
| 456-132 | 324 |  |
|  |  |  |
| 62-18 | 44 |  |
| 317-126 | 191 |  |
| 632-278 | 354 |  |
| **Total subtraction** | | **/6** |

**Written multiplication**

| Question | Answer | Score |
| --- | --- | --- |
| 54x2=108 | 108 |  |
|  |  |  |
| 25x8=200 | 200 |  |
| 34x16=544 | 544 |  |
| 429x53=22737 | 22737 |  |
| 618x203=125454 | 125454 |  |
| **Total Multiplication** | | **/5** |

# Total Written Operations: /17
